# Nanoparticle Effects on Human Platelets *in Vitro*: A Comparison between PAMAM and Triazine Dendrimers

**DOI:** 10.3390/molecules21040428

**Published:** 2016-03-29

**Authors:** Alan E. Enciso, Barry Neun, Jamie Rodriguez, Amalendu P. Ranjan, Marina A. Dobrovolskaia, Eric E. Simanek

**Affiliations:** 1 Department of Chemistry & Biochemistry, Texas Christian University, Fort Worth, TX 76129, USA; a.encisobarros@tcu.edu; 2 Nanotechnology Characterization Lab, Frederick National Laboratory for Cancer Research, Frederick, MD 21702, USA; neunb@mail.nih.gov (B.N.); rodriguezjc@mail.nih.gov (J.R.); 3 Department of Molecular and Medical Genetics & Institute of Cancer Research, University of North Texas Health Science Center, Fort Worth, TX 76109, USA; amalendu.ranjan@unthsc.edu

**Keywords:** dendrimer, triazine, PAMAM, platelet, biocompatibility

## Abstract

Triazine and PAMAM dendrimers of similar size and number of cationic surface groups were compared for their ability to promote platelet aggregation. Triazine dendrimers (G3, G5 and G7) varied in molecular weight from 8 kDa–130 kDa and in surface groups 16–256. PAMAM dendrimers selected for comparison included G3 (7 kDa, 32 surface groups) and G6 (58 kDa, 256 surface groups). The treatment of human platelet-rich plasma (PRP) with low generation triazine dendrimers (0.01–1 µM) did not show any significant effect in human platelet aggregation *in vitro*; however, the treatment of PRP with larger generations promotes an effective aggregation. These results are in agreement with studies performed with PAMAM dendrimers, where large generations promote aggregation. Triazine dendrimers promote aggregation less aggressively than PAMAM dendrimers, a factor attributed to differences in cationic charge or the formation of supramolecular assemblies of dendrimers.

## 1. Introduction

Dendrimers are nanosized, hyperbranched polymers with low polydispersity that are amenable to synthetic manipulation [1,2,3,4]. While not yet realized, the potential for dendrimers to act as drug delivery vehicles has been long appreciated [5,6,7,8,9]. Our efforts, like others, rely on intravenous administration of such vehicles [10,11,12,13]. This strategy promotes interactions of these protein-sized architectures with endogenous proteins, cells, and aggregates such as lipoproteins. A common design strategy for the application of these materials relies on dendrimers to promote sustained systematic distribution by retention in the circulation. Accordingly, dendrimers will experience increased exposure time with components of the blood stream. Assessment of biocompatibility typically commences with studies of dendrimer interactions with specific components of the circulatory system. Interactions with platelets represent a common starting point for such studies. Platelets are abundant in the vasculature and are sensitive to changes in blood microenvironment [14]. Platelet aggregation is a critical step in the clotting cascade [15]. Agents that promote or attenuate this behavior are of clinical interest [16,17,18,19,20,21]. While nanomaterials have demonstrated these abilities, such effects are “off-target” if the goal is drug delivery [22].

**Chart 1:**
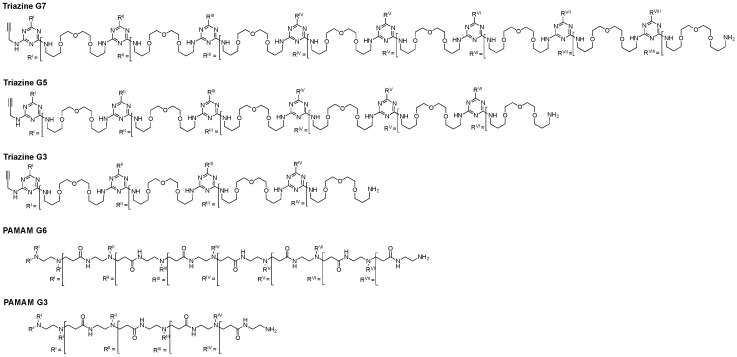
Structures of the triazine and PAMAM dendrimers examined.

Historically, PAMAM dendrimers have served as a benchmark for other classes of dendrimers. Here, we compare PAMAM standards with triazine dendrimers [23]. Earlier structure activity relationship studies with PAMAM dendrimers revealed that dendrimer size, zeta potential, and density of the surface amines are responsible for the dendrimer interaction with, and activation of, platelets [14,22,23]. The mechanism of activation has been attributed to the disturbance of the cellular membrane integrity [23]. However, the composition of triazine dendrimers is different to PAMAM dendrimers (Chart 1). The branching point for triazine dendrimers is the rigid, aromatic triazine ring. In contrast, PAMAM branch from a tertiary amine. Differences in flexibility, hydrophilicity, size, and basicity are apparent. For the studies described here, the triazine rings are interconnected with a tetraethyleneglycol group to promote water solubility. For simplicity, the molecules will be indicated by class and generation, abbreviated “G.”

## 2. Results and Discussion

### 2.1. Zeta Potential

Triazine and PAMAM dendrimers are intrinsically different in composition. However, at large generation both are perceived to exist as globular spheres resembling proteins with biophysical properties that is attributed to their surface chemistry [24,25,26,27,28]. The zeta-potentials measured for these molecules at near neutral pH using 10 mM phosphate buffer (and 136 mM NaCl) show that they are all cationic as expected, but the triazines bear less charge than the corresponding PAMAM, even when surface group numbers are identical. At 10 mM buffer without added salt, the triazines zeta-potentials are 32 mV, 22 mV, and 26 mV, respectively for G3, G5, and G7. This difference could be attributed to the preponderance of interior, tertiary amines of the PAMAM whose pKa are approximately 10 compared with the triazine sites with pKas of approximately 4.5.

Charge density might seem similar given similar charges and monomer size, but these dendrimers aggregate in solution. The triazines monomers of the dimensions shown in Table 1 are in equilibrium with multimeric aggregates measuring hundreds of nanometers. The data presented in Table 1 are summarized from our earlier studies [23,29,30]. Indeed, this behavior underscores the importance of these studies on triazines given their intended uses. While similar in branching architecture and surface chemistry, dendrimers within and across classes of composition can potentially display different behaviors in complex environments like the vasculature.

### 2.2. Platelet Aggregation

**Figure 1:**
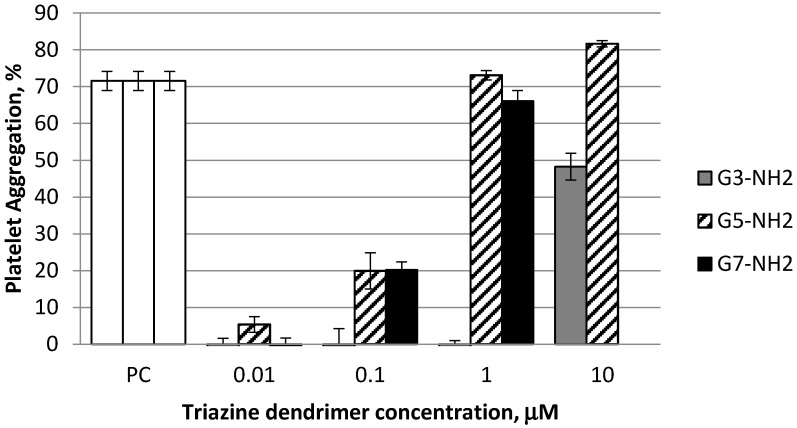
Induction of platelet aggregation by amine terminated triazine dendrimers is size-dependent. Different concentrations of generations 3, 5, and 7 of triazine dendrimers with amine terminal groups that were incubated with human platelet reach plasma for 15 min at 37 °C. After that, platelet count was performed using a Z2 cell and particle counter. Platelet count in the test samples was compared to that in the negative control sample to calculate percent platelet aggregation. Collagen was used as an assay positive control (PC). Shown is mean ± SD (*n* = 3).

Incubating triazine G3, G5, and G7 dendrimers in plate-rich plasma leads to platelet activation, which culminates in platelet aggregation. Using four different concentrations (0.01 µM, 0.10 µM, 1 µM, and 10 µM), we find that activation occurs in a dose-dependent and size-dependent manner. Triazine G5 and G7 dendrimers activate platelets more than triazine G3 dendrimers (Figure 1). Due to the insufficient quantity of material, the triazine G7 dendrimer was not assayed at the highest concentration. These results are in agreement with studies performed with PAMAM dendrimers that show increasing activation with increasing size [23].

**Figure 2:**
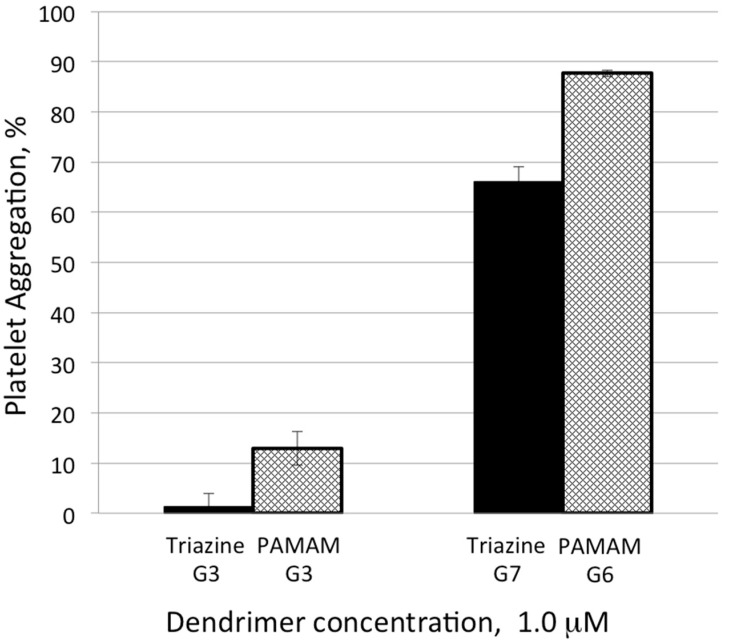
Cationic triazine dendrimers are less reactive with human platelets than cationic PAMAM dendrimers of similar size. G3- and G7-amine-terminated triazine dendrimers and G3- and G6-amine terminated PAMAM dendrimers at equivalent molar concentrations were incubated with human platelet reach plasma for 15 min at 37 °C. After that, platelet count was performed using a Z2 cell and particle counter equipped with a 5-μm aperture tube. Platelet count in the test samples was compared to that in the negative control sample to calculate percent platelet aggregation. Collagen was used as an assay positive control (data not shown). Shown is mean ± SD (*n* = 3).

In a comparative test, G3 and G7 triazine dendrimers (1 µM) are seen to be less aggressive at platelet aggregation compared to their PAMAM counterparts of similar generation possessing similar physicochemical properties (G3 and G6) (Figure 2). Due to the absence of well characterized G7 PAMAM dendrimer, we used G6 PAMAM dendrimer in this study. Since platelet aggregation induction by PAMAM dendrimer increases with their size (23), it is reasonable to expect that platelet aggregation by G7 PAMAM dendrimer will be even stronger than that observed with G6. Therefore, the difference between G7 of Triazine and G7 PAMAM dendrimers would be even greater than that reported in this study.

## 3. Materials and Methods

### 3.1. Reagents

DPBS (Ca^2+^/Mg^2+^ free), poly-l-lysine hydrobromide, polymyxin B, 2MeSAMP, and 1,10-phenantroline were from Sigma-Aldrich (St. Louis, MO, USA). G3 and G6 PAMAM dendrimers with amine surface were from Dendritic Nanotechnologies Inc. (Mount Pleasant, MI, USA). Collagen was purchased from Helena Laboratories (Beaumont, TX, USA). Triazine dendrimers were synthesized according to procedures previously reported [31,32].

### 3.2. Research Donor Blood

Healthy volunteer blood specimens were drawn under NCI-Frederick Protocol OH99-C-N046. Blood was collected in BD vacutainer tubes containing sodium citrate as an anticoagulant. To avoid individual variability, specimens from at least three donors were pooled.

### 3.3. Platelet Aggregation

To study particles effects on platelet aggregation, whole blood was centrifuged 8 min at 200× *g* in order to obtain platelet rich plasma (PRP). PRP was treated with nanoparticles, PBS (negative control), or collagen (positive control) for 15 min at 37 °C. After that, single platelet count was conducted using a Z2 counter and a size analyzer (Beckman Coulter, Brea, CA, USA). Difference in single platelet count between negative control and test samples was used to calculate percent platelet aggregation. Additional control included incubation of platelet poor plasma and PBS with particles and analyzing these samples on the instrument. These controls were used to monitor potential particle aggregation in the presence of plasma proteins to avoid false-negative results. Detailed protocol is available [33]. To study potential particle contamination with endotoxin, the test samples were analyzed by turbidity LAL assay according to the protocol published [34]. Endotoxin was not detected in any test samples at concentrations used in the platelet aggregation assay.

### 3.4. Zeta Potential Measurements

The zeta potential measurement of all the generations of dendrimers were conducted with Zetasizer Nano-ZS (Malvern Instruments, Westborough, MA, USA). Samples were suspended in 1× PBS pH 7.4 (with and without salt) and placed in disposable folded capillary cells DTS1070 (Malvern Instruments, Westborough, MA, USA). The electrophoretic mobility of the samples were measured in an applied electric field. Twelve zeta potential measurements were collected for each run, and the results were averaged. The Zeta potential value was calculated directly from the Smoluchowski model using the Malvern software.

## 4. Conclusions

Consistent with Tomalia’s nanoperiodicity hypothesis [35,36,37], both triazine and PAMAM dendrimers activate platelets in a size- and charge-dependent manner in that dendrimers of earlier generations (G3) exhibit lower potency in activating platelets than their higher generation (G6 and G7) counterparts. Dendrimers of earlier generation are smaller in hydrodynamic size and have lower number of surface amines. The data obtained for PAMAM dendrimers in our study is similar to the earlier reports [22,23]. Of interest, herein we demonstrated that Triazine dendrimers are less potent than their PAMAM counterparts. The diminished activity displayed by the triazine dendrimers is likely attributed to the lessor density of surface amines. That is, triazine dendrimers due to the lack of interior groups protonated near neutral pH. Systemic administration of cationic PAMAM dendrimers results in consumptive coagulopathy, a thrombogenic disorder halting the use of these particles as drug delivery vehicles [22]. The data presented in our study highlight less thrombogenic properties of cationic triazine dendrimers and warrant further investigation of these particles as potential drug carriers.

## Figures and Tables

**Table 1:** Comparison of triazine and PAMAM dendrimers.

Dendrimer	Z-Potential (mV)	Monomer Size (nm)	Peripheral Amines	Internal Tertiary Amines	MW (Da)
G3-Triazine	23.1	3.7	16	-	7785
G3-PAMAM	43.3	3.1	32	30	6910
G5-Triazine	16.5	8.0	64	-	33 K
G6-PAMAM	46.2	7.5	256	254	58 K
G7-Triazine	19.8	13.7	256	-	130 K
